# Natural history of hypertrophic cardiomyopathy in cats from rehoming centers: The CatScan II study

**DOI:** 10.1111/jvim.16576

**Published:** 2022-10-31

**Authors:** Jose Novo Matos, Jessie Rose Payne, Joonbum Seo, Virginia Luis Fuentes

**Affiliations:** ^1^ Clinical Science and Services Royal Veterinary College Hertfordshire UK; ^2^ Langford Vets Small Animal Referral Hospital University of Bristol Bristol UK; ^3^ Animal Referral Centre Auckland New Zealand; ^4^ School of Veterinary Science Massey University Palmerston North New Zealand

**Keywords:** dynamic left ventricular outflow tract obstruction, left atrial fractional shortening, left atrial function, predictors of HCM, shelter cats, systolic anterior motion

## Abstract

**Background:**

The natural history of hypertrophic cardiomyopathy (HCM) in cats has been mainly studied in cats referred for suspected heart disease, which can skew the results towards cats with clinical signs. Few data are available on factors associated with development of HCM in cats.

**Hypotheses:**

(1) Clinical variables can predict which cats will develop HCM; (2) HCM in cats not referred for suspected heart disease is associated with a low rate of cardiovascular events.

**Animals:**

One hundred seven cats from rehoming centers without a history of clinical signs of cardiac or systemic disease at the time of adoption.

**Methods:**

Prospective longitudinal study. After rehoming, shelter cats were reexamined for serial echocardiograms. Cox regression analysis was used to identify predictors of development of HCM in cats that were normal at baseline. Adverse cardiovascular events including heart failure, thromboembolism, or sudden death were recorded.

**Results:**

Cats were monitored for a median of 5.6 [1.2‐9.2] years. At baseline, 68/107 cats were normal, 18/107 were equivocal and 21/107 had HCM. Nineteen cats developed HCM during the study period. The factors at baseline associated with increased hazard of developing HCM were lower left atrial fractional shortening, higher left ventricular fractional shortening, and higher body weight. Cardiovascular events were observed in 21% of cats with HCM.

**Conclusions and Clinical Importance:**

Cardiovascular events were common in cats with HCM from a rehoming center study sample. Lower left atrial systolic function appears to precede overt HCM.

**Abbreviations**
2D echo: 2‐dimensional echocardiography
ATE: arterial thromboembolism
CHF: congestive heart failure
G+/LVH‐: genotype positive/phenotype negative
HCM + SAM: hypertrophic cardiomyopathy with systolic anterior motion of the mitral valve
HCM: hypertrophic cardiomyopathy
IVS: interventricular septum
LA: left atrial
LA/Ao: ratio of the left atrium to aorta
LAFS%: left atrial fractional shortening
LV: left ventricular
LVFS%: left ventricular fractional shortening
LVFW: left ventricular free wall
LVH: left ventricular hypertrophy
LVIDd: left ventricular internal diameter in diastole
LVOTO: left ventricular outflow tract obstruction
LVWT: left ventricular wall thickness
RPLA: right parasternal long‐axis
RPSA: right parasternal short‐axis
SAM: systolic anterior motion
SD: sudden cardiac death

## 1 INTRODUCTION

Most reports of the natural history of hypertrophic cardiomyopathy (HCM) in humans^1^, ^2^, ^3^ and cats^4^, ^5^, ^6^, ^7^, ^8^ have been based on studies performed at referral centers. In people, referral cohorts can be biased towards patients suspected of heart disease, skewing the sampling to higher risk patients and more severe forms of HCM, which can lead to overestimation of disease malignancy.^9^, ^10^, ^11^ Human studies of HCM in nonreferral (general) centers, which are more likely to reflect the true natural history of this disease, consistently have lower rates of morbidity and death and a more benign prognosis than people diagnosed with HCM at at referral centers.^9^, ^10^, ^11^, ^12^, ^13^ While some human HCM patients experience serious complications, the majority will have an uneventful clinical course and normal longevity.^12^, ^13^

The REVEAL study^8^ evaluated the natural history of subclinical HCM in a large study sample of cats from referral centers worldwide. This is the largest veterinary study to date evaluating the prognosis of cats with subclinical HCM, describing an overall incidence of cardiovascular events and death of approximately 30%.

In people, changes in left ventricular (LV) function and geometry precede LV hypertrophy (LVH), suggesting that LVH is not the first manifestation of HCM.^14^ Certain clinical characteristics, such LV ejection fraction, elongated mitral leaflets and ECG abnormalities, are suggested to predict development of LVH (overt HCM).^15^, ^16^

The natural history and prognosis of HCM in cats not referred for suspected heart disease have not been reported. Furthermore, there are scarce data assessing clinical or echocardiographic factors that might predict the development of HCM in cats. In the present longitudinal study, we serially examined a study sample of cats from rehoming centers in the UK. Our hypotheses were: (1) Clinical variables can predict which cats will develop an HCM phenotype; (2) HCM in cats not referred for a suspected heart disease is associated with a low rate of cardiovascular events. The primary aims of this study were: (1) to determine whether any clinical variables are associated with increased risk of developing HCM by studying cats that were normal at baseline and developed HCM during the study period; (2) to assess the incidence of cardiovascular events including congestive heart failure (CHF), arterial thromboembolism (ATE) and sudden cardiac death (SD) in cats with HCM from rehoming centers.

## 2 MATERIALS AND METHODS

This was a prospective, longitudinal study of the natural history of HCM in cats from rehoming centers (CatScan II study). This study was the longitudinal arm of a cross‐sectional study (CatScan I study).^17^ The cross‐sectional study collated clinical and echocardiographic data from 780 cats without a history of clinical signs of cardiac or systemic disease at 2 UK rehoming centers. At the time of rehoming, new owners adopting participating cats were offered free periodic cardiac evaluations. The owners of 570/780 cats agreed and gave written consent for their cat to be enrolled in the CatScan II study. Cats enrolled in the longitudinal arm of the study included both cats with structurally normal hearts and cats with subclinical HCM (stage B).^18^ The cats had no history of clinical signs of cardiac or systemic disease at the time of adoption.

Throughout November 2015 and May 2020, the owners of the 570 cats enrolled in the longitudinal study were invited by e‐mail to bring their cats for an echocardiographic reexamination. Reexaminations took place at the 2 original rehoming centers, local veterinary practices or at our referral hospital. Only cats with at least 1 follow‐up echocardiographic examination (ie, a minimum of 2 scans in total) were included in the present study.

### 2.1 Cats lost to follow‐up

There were 459/570 cats initially enrolled in the longitudinal study that were subsequently lost to follow‐up. A summary of the reasons for losing cats to follow‐up is described in Table 1.

**TABLE 1:** Cats lost to follow‐up

	N
Cats initially enrolled in CatScan II	570
Cats lost to follow‐up	459
Reason for withdrawal	
Owner contact details unavailable or no reply to emails	287
Owner unable to attend appointments	70
Died	62
Noncardiac death^a^	40
Unknown causes	12
Cardiac death (3 ATE, 3 CHF, 4 SD)	10
Owner unwilling to participate	40

Abbreviations: ATE, arterial thromboembolism; CHF, congestive heart failure; SD, sudden cardiac death.

^a^ Noncardiac deaths: cancer (8 cats), chronic kidney disease (8 cats), miscellaneous systemic diseases (13 cats), road traffic accident (11 cats).

### 2.2 Cats enrolled in the longitudinal study

**FIGURE 1:**
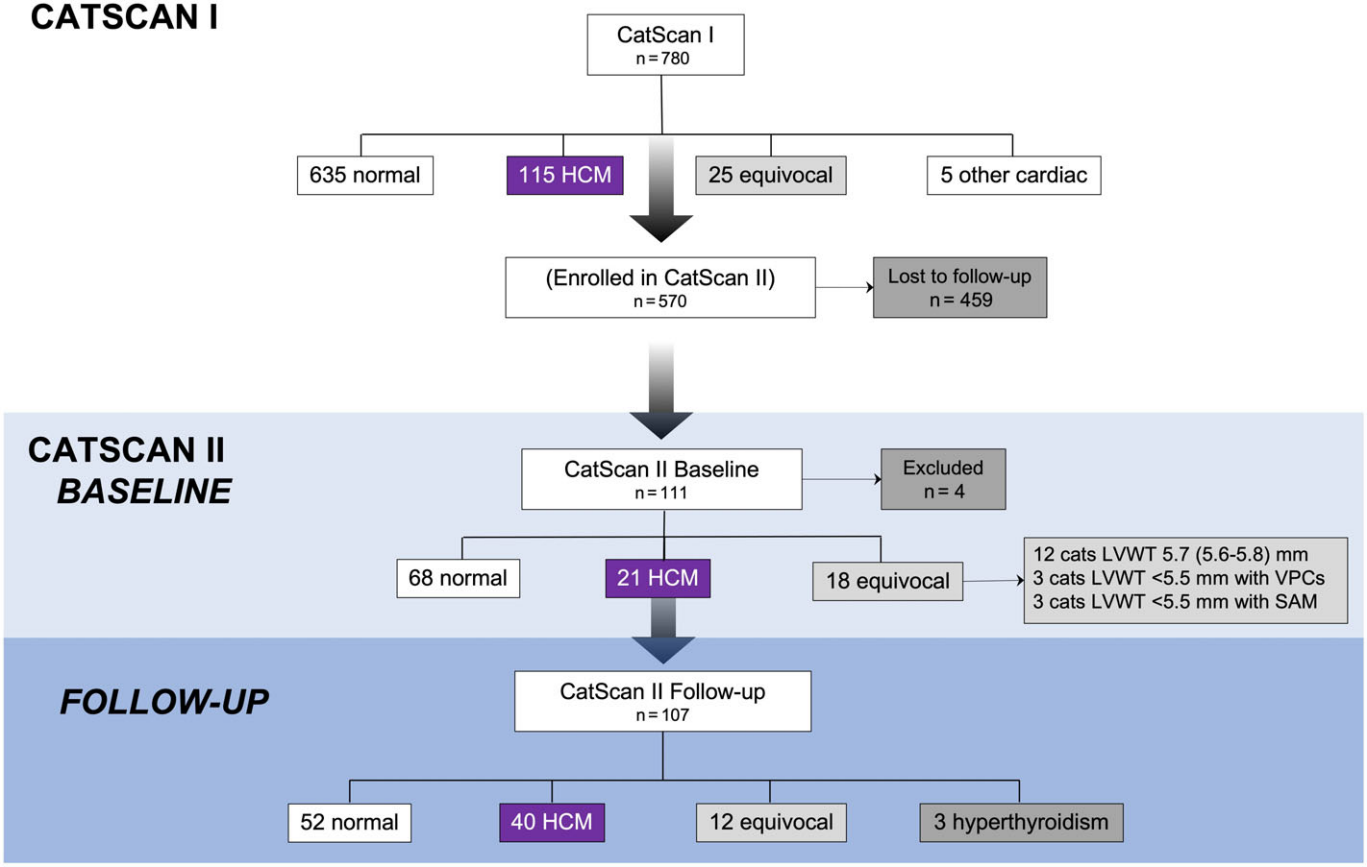
Summary of the cats enrolled in longitudinal study

One hundred eleven cats were reexamined after rehoming. The scan and clinical data were lost from 3/111 cats, and scan quality was inadequate in 1 cat, thus the study sample comprised 107 cats. At each visit, physical examination, blood pressure measurement, and echocardiography were performed. Additionally, N‐terminal pro B‐type natriuretic peptide was measured in cats with echocardiographic abnormalities, and an ECG was performed if an arrhythmia was detected. A summary of the cats enrolled in the longitudinal study is described in Figure 1.

### 2.3 Echocardiographic data

At the time of data analysis, 1 observer Jose Novo Matos reviewed and remeasured the echocardiograms of all 107 cats. The images of 10 initial scans (baseline) were not available for review, so the measurements performed at the time of image acquisition were used for data analysis. The initial (baseline) and final (follow‐up) scans were used for final data analysis, except for Cox regression analysis where the time to the first scan where HCM (event) was diagnosed was analyzed.

Echocardiographic examinations were performed using an Esaote MyLab30 (MyLab 30 Gold, Esaote UK, Cambridge, UK), Vivid E9 or Vivid I ultrasound machine (GE Systems, Hatfield, Hertfordshire, UK) with a 7.5 or 12 MHz phased‐array transducer. Standard right parasternal 2‐dimensional echocardiographic (2D echo) views were acquired and measurements performed in recorded loops.^17^ None of the cats were sedated.

Left ventricular (LV) wall thickness and chamber dimensions and left atrial (LA) dimensions were measured from 2D echo views. Left ventricular free wall (LVFW) and interventricular septum (IVS) thicknesses were measured at end‐diastole from a right parasternal long‐axis 4‐ or 5‐chambered view (RPLA) and a short‐axis view (RPSA) at the papillary muscle level, using a leading edge to leading edge technique. The thickest end‐diastolic segment of the IVS and LVFW was measured from 3 different cardiac cycles and averaged separately for the RPLA and RPSA view. The greatest LV wall thickness from all 4 measurements (RPLA IVS, RPLA LVFW, RPSA IVS, RPSA LVFW) was the value used for final data analysis, and defined as maximal left ventricular wall thickness (LVWT).

All cardiac chambers (LV and LA dimensions) were measured with an inner edge to inner edge technique at the interface between the blood pool and myocardial wall.^19^ Left ventricular internal diameter in diastole (LVIDd) was measured at end‐diastole, from a RPLA and RPSA view at the level of the chordae tendineae on 3 different cardiac cycles in each view. The averaged LVIDd in each of these 2 views was recorded, and the highest value was used for final data analysis. Left atrial dimension was measured as the ratio of the left atrium to aorta (LA/Ao) measured from a RPSA view at the heart base at end‐ventricular systole,^20^ and the LA diameter measured as the maximal cranial‐caudal LA dimension in a RPLA 4‐chamber view at end‐ventricular systole.^21^

Left atrial (LAFS%) and left ventricular (LVFS%) fractional shortening were both measured by 2D‐guided M‐Mode from a RPSA at the heart base^20^ and at the papillary muscle level, respectively.

The presence of systolic anterior motion of the mitral valve (SAM) was assessed on 2D echo and color Doppler from a RPLA 5‐chambered view, as systolic motion of the tip of the anterior mitral valve leaflet towards the IVS with turbulent flow in the left ventricular outflow tract.

Hypertrophic cardiomyopathy (HCM) was defined as LVWT ≥6 mm, in the absence of systemic hypertension (systolic blood pressure >160 mm Hg)^22^ and hyperthyroidism. All cats >8 years of age had serum total thyroxine measured at baseline and follow‐up. Cats with LVWT ≥6 mm and SAM were classified as HCM with SAM (HCM + SAM). “Equivocal” for cardiomyopathy was defined as LVWT 5.5‐5.9 mm or LVWT <5.5 mm with arrhythmias or SAM. Normal LVWT was defined as <5.5 mm.

### 2.4 Clinical data

At each follow‐up visit data were collected: age, body weight (BW), body condition score (assessed on a scale of 1‐9), heart rate (HR), presence of a murmur, gallop or arrhythmia and presence of clinical signs of systemic or cardiac disease. Systolic blood pressure was determined noninvasively using a Doppler device.

### 2.5 Outcome data

Cardiovascular events (CHF, ATE, or SD) during the study period were recorded. Sudden death was defined as an unexpected death without any clinical signs or known trauma in the preceding 24 hours.^23^ The incidence of cardiovascular events was calculated for the exact time at risk^24^ in the HCM study sample. Incidence is expressed as number of events per 1000 cat years.

### 2.6 Statistical analysis

Data were tested for normality graphically and by a Shapiro‐Wilk test. Normally distributed data are reported as mean (95% confidence interval) and nonnormally distributed data as median [range]. Categorical data are presented as frequency and percentage. Within‐group comparisons (baseline vs follow‐up) were carried out by Wilcoxon signed‐rank test or paired *t*‐test as appropriate for continuous variables, and McNemar's test for categorical variables. Mann‐Whitney *U*‐test or students' independent *t*‐test and Pearson chi‐squared or Fisher's exact were used for between‐group comparisons of continuous and categorical variables, respectively. In cases where data were missing, the number of cats available for analysis is reported.

Demographic baseline characteristics of the longitudinal study sample were compared with the original cross‐sectional study sample using a chi‐square goodness of fit test.

Baseline characteristics of cats that were normal or equivocal and developed HCM during the study (normal/equivocal‐HCM) were compared to cats remaining normal or equivocal over the study period (normal/equivocal‐normal/equivocal) in order to identify predictors of development of HCM. Cats that developed hyperthyroidism were excluded. This analysis was run twice using 2 LVWT cut‐offs to define HCM: LVWT ≥5.5 mm and LVWT ≥6 mm. Potential risk factors for the development of HCM were assessed by Kaplan‐Meier curves and compared by a Logrank (Mantel‐Cox) test, and by univariable Cox proportional hazards analysis. Multivariable analysis was run for variables significant at *P* < .1 in a backward stepwise manner. Hazard ratios and 95% confidence interval were calculated. Proportional hazard assumption was assessed by inspection of Kaplan‐Meier curves, by including a time‐dependent interaction variable into the model and by assessment of Schoenfeld residuals. Collinearity was evaluated by assessing correlations between variables in the multivariable model. Overall model fit was analyzed via graphical assessment of residuals and by Akaike's Information Criterion. The odds of developing HCM according to LAFS% were calculated as follows HR/[1 + HR].^25^

Percentage change of LVWT/per year over the study period was calculated for the cats with normal echocardiograms at baseline (LVWT <6 mm) as follows:

%ΔLVWT/year = (LVWT^follow‐up^ − LVWT^baseline^)/(LVWT^follow‐up^ * 100)/follow‐up time. Spearman correlation analysis was used to evaluate the association between %ΔLVWT/year and age at baseline; systolic blood pressure change (ΔSBP = SBP^follow‐up^ − SBP^baseline^); and BW change (ΔBW = BW^follow‐up^ − BW^baseline^).

Intraobserver measurement agreement and variability for LVWT and LA%FS were quantified by intraclass correlation coefficient (ICC) and coefficient of variation (%CV). The observer Jose Novo Matos measured 10 randomly selected echocardiograms (5 cats with HCM and 5 normal cats) 4 weeks apart. An ICC > 0.75 and %CV < 10% were considered to indicate excellent measurement agreement and low variability, respectively.

*P* values <.05 were considered statistically significant. Statistical analysis was performed by commercially available software (SPSS 25‐26, 2018‐2019; GraphPad Prism 7, 2016).

## 3 RESULTS

### 3.1 Whole study sample

The longitudinal study sample comprised 107 cats aged 2.8 [0.1‐13] years at baseline monitored over 5.6 [1.2‐9.2] years. Cats had a median of 2 [2–5] scans, that is, 1 baseline and 1 follow‐up scan. The majority were nonpedigree (104/107, 97%), with 1 Burmese, 1 Ragdoll and 1 Russian White cat. Sex was equally represented (53/107 females, 49.5%). Overall, cats at the follow‐up examination were heavier, had a higher body condition score, higher blood pressure, lower HR, thicker LVWT, and greater LVIDd, LA size and LVFS% (Table 2).

**TABLE 2:** Baseline and follow‐up characteristics of the whole study sample

	N	Baseline	Follow‐up	*P* value
Demographics				
Age (y)		2.8 [0.1‐13.0]	**8.7 [**3.0‐17]	–
Weight (kg)	71	3.7 [1.3‐7.9]	**4.7** [2.8‐7.2]	**<.001**
BCS (/9)	83	5 [4‐8]	**5** [3‐8]	**<.001**
Murmur (%)	100	50 (50%)	57 (57%)	.27
Blood pressure	56	121 [100‐167]	**130** [103‐190]	**.002**
Heart rate (bpm)	72	210 (201‐219)	**192** (184‐199)	**.001**
Echocardiography				
LVWT (mm)		4.8 [2.8‐8.2]	**5.5** [3.5‐8.8]	**<.001**
IVSd (mm)	106	4.7 [2.8‐7.0]	**5.3** [3.5‐7.6]	**<.001**
LVFWd (mm)		4.5 [2.4‐8.0]	**5** [3.0‐8.8]	**<.001**
LVIDd (mm)	98	14.5 (14.2‐14.9)	**15.6** (15.2‐15.9)	**<.001**
LAD (mm)	91	13.6 [10‐18.2]	**15.5** [12.1‐24.4]	**<.001**
LA/Ao	102	1.4 [1.0‐1.7]	**1.5** [1.1‐2.5]	**<.001**
LAFS%	88	26 [14‐39]	26 [5.9‐38]	.1
LVFS%	40	35 [27‐53]	**45** [26‐58]	**<.001**
HCM (%)		21 (19.6%)	**40 (37.4%)**	**<.001**
HCM + SAM (%)		7/21 (33.3%)	16/40 (40%)	

*Note*: Comparisons between baseline and follow‐up were performed by Wilcoxon signed‐rank test or paired *t*‐test for nonnormally and normally distributed continuous variables, respectively. McNemar's test was used for categorical variables. Statistically significant differences (*P* < .05) are highlighted in bold.

Abbreviations: BCS, body condition score; HCM, hypertrophic cardiomyopathy; HCM + SAM, HCM with systolic anterior motion; IVSd, interventricular septum at end‐diastole; LAD, left atrial diameter; LA/Ao, ratio of the left atrial to aortic ratio; LAFS%, left atrial fractional shortening; LVFS%, left ventricular fractional shortening; LVIDd, left ventricular internal diameter at end‐diastole; LVFWd, left ventricular free wall at end‐diastole; LVWT, maximal left ventricular wall thickness; SAM, systolic anterior motion.

#### 3.1.1 Changes in cardiac phenotype over time

**FIGURE 2:**
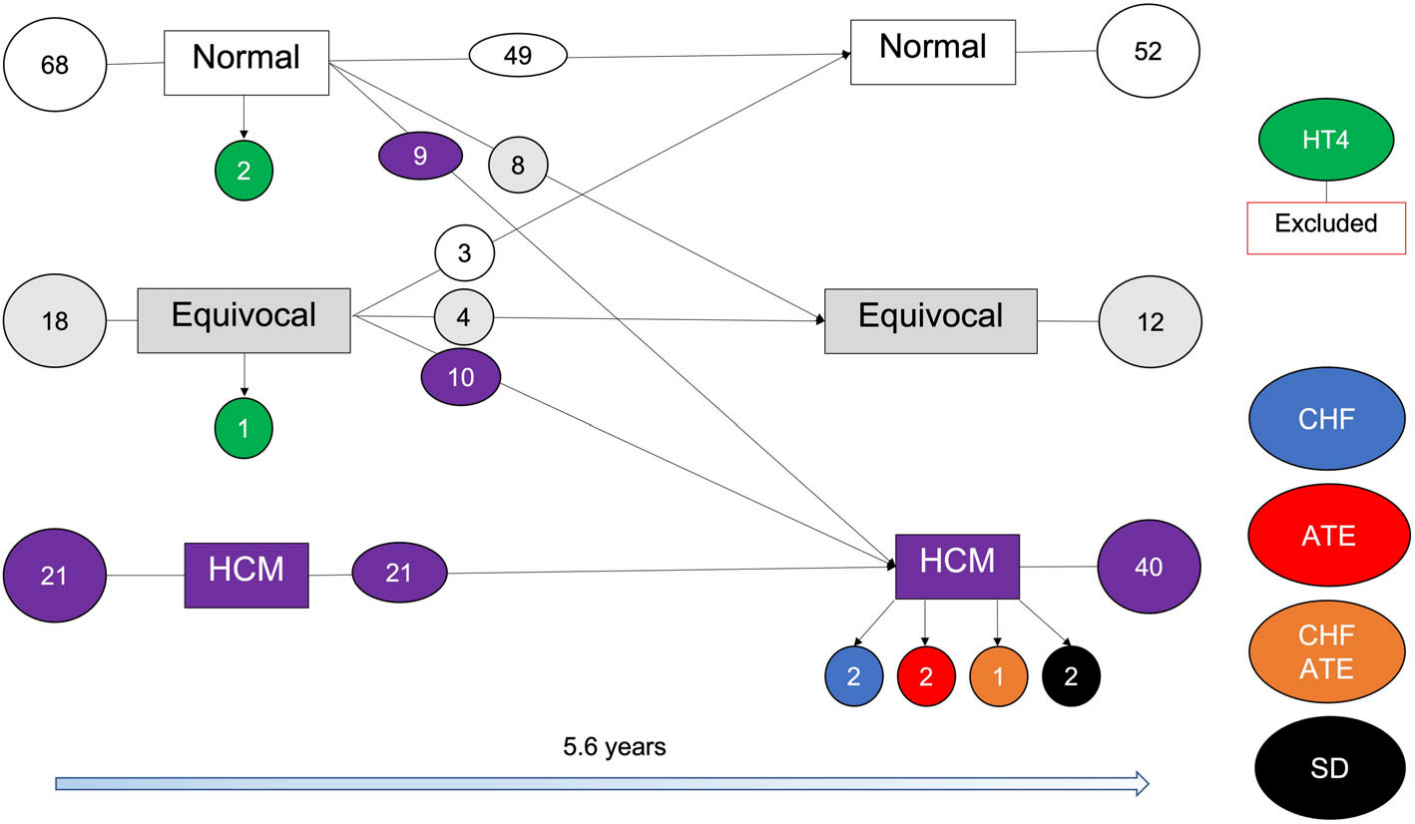
Changes in cardiac phenotype from the initial baseline assessment to the final follow‐up

At baseline, 68/107 (63.6%) cats were normal, 18/107 (16.8%) were equivocal and 21/107 (19.6%) had HCM (Figure 2). In 12/18 cats equivocal for cardiomyopathy, LVWT was between 5.5‐5.9 mm (5.7 [5.6‐5.8] mm). The other 6/18 cats were classified as equivocal because of isolated ventricular premature complexes (3/6) or SAM (3/6) with normal LVWT [3.8‐5.4] mm. At follow‐up, 52/107 (48.9%) cats were normal and 12/107 (11.2%) cats were equivocal. Thirty (30/107) cats changed classification (Figure 2). The 3 cats with arrhythmias and normal LVWT at baseline still had normal LVWT at a median follow‐up of 7.5 [6.6‐7.6] years, and no arrhythmias were detected on the follow‐up exam. There was an increase in HCM prevalence from 19.6% to 37.4% at follow‐up (*P* < .001). Three cats developed hyperthyroidism during the study, 2 of which had normal LVWT at baseline (<5.5 mm) and 1 was equivocal. These cats were excluded from the follow‐up analyses. A summary of the demographic and echocardiographic characteristics of the whole study sample at baseline and follow‐up is described in Table 2, and Figures 1 and 2.

### 3.2 Cats with HCM at initial evaluation

Baseline and follow‐up characteristics of cats with HCM at baseline are summarized in Table 3. Mean follow‐up time was 4.4 (3.4‐5.5) years. There was no unidirectional change in LVWT over time, whereas LA size and LVIDd increased. The proportion of cats with SAM did not change, with a prevalence of 33% (7/21) at baseline and 28% (6/21) at follow‐up (*P* = .7). Two cats developed ATE and 1 developed CHF. Two cats developed regional LV wall thinning and hypokinesis over time, such as would be seen with a transmural myocardial infarct. One cat with asymmetric LVH (LVFW hypertrophy) at baseline had progressive LVFW thinning with severe LVFW hypokinesis. This cat had 5 follow‐up scans over 7 years, with LVFW thickness decreasing from 7.0 mm at baseline to 2.1 mm at the final follow‐up, while the IVS was 4.9 mm at baseline and 5.4 mm at follow‐up. This cat remained asymptomatic during the study period. Another cat with concentric LVH at baseline showed thinning of the interventricular septum at a follow‐up scan 1.9 years later. The IVS measured 6.3 mm at baseline and 3.5 mm at follow‐up, while the LVFW remained thickened, measuring 8.0 mm at baseline and 7.8 mm at follow‐up. This cat developed ATE. Besides these 2 cats, no other cats with HCM had changes in LVH distribution or LV remodeling.

**TABLE 3:** Baseline and follow‐up characteristics of 21 cats with hypertrophic cardiomyopathy (HCM) at initial evaluation

	n	Baseline	Follow‐up	*P* value
Demographics				
Age (y)		6.4 (4.8‐8.0)	**10.8** (9.7‐12)	–
Weight (kg)	12	4.6 (3.8‐5.5)	4.7 (4.3‐5.1)	.8
BCS (/9)	14	5 [4‐7]	6 [3‐7]	.6
Murmur (%)	17	15 (88.2%)	16 (94.1%)	1.0
Blood pressure	13	128 (115‐141)	**140.4** (125.3‐155.5)	**.04**
Heart rate (bpm)	14	216.1 (196‐236)	200 (183‐217)	.17
Echocardiography				
LVWT (mm)		6.3 [6‐8.2]	6.5 [5.4‐7.8]	.2
IVSd (mm)		6.1 [4.9‐8.2]	6.4 [3.5‐7.8]	.5
LVFWd (mm)		5.8 (5.4‐6.2)	5.8 (5.3‐6.3)	.9
LVIDd (mm)	19	13.9 (13.1‐14.6)	**15.1** (14.1‐16.0)	**.02**
LAD (mm)	19	14.7 (13.7‐15.7)	**16.5** (15.4‐17.6)	**.002**
LA/Ao	19	1.3 (1.3‐1.4)	**1.5** (1.3‐1.6)	**.04**
LAFS%	18	24.3 (22.7‐26)	23.6 (20.6‐26.7)	.6
LVFS%	6	39.5 (31.2‐47.8)	44.3 (34.1‐54.6)	.2
HCM + SAM (%)		7 (33%)	6 (29%)	.7

*Note*: Comparisons between baseline and follow‐up were performed by Wilcoxon signed‐rank test or paired *t*‐test for nonnormally and normally distributed continuous variables, respectively. McNemar's test was used for categorical variables. Statistically significant differences (*P* < .05) are highlighted in bold.

Abbreviations: See Table 1.

### 3.3 Cats that developed HCM during the study (normal/equivocal‐HCM)

Eighty‐three cats were normal or equivocal at baseline and did not develop hyperthyroidism over time (Figure 2). At follow‐up, 64/83 remained normal/equivocal and 19/83 developed HCM. Of these 19 cats, 10 (53%) had SAM and 11 (58%) had LA dilation (LA/Ao 1.8 [1.6‐2.5]). N‐terminal pro B‐type natriuretic peptide results were available in 12/19 cats at the time of HCM diagnosis, with a median value of 400 (198‐602) pmol/L. Cardiovascular events were recorded for 4 cats: 1/4 CHF, 1/4 both CHF and ATE and 2/4 SD.

Comparison of baseline and follow‐up characteristics of cats developing HCM (normal/equivocal‐HCM) vs cats remaining free of HCM (normal/equivocal‐normal/equivocal) during the study period are summarized in Table 4. The follow‐up time between groups was similar (5.4 [1.2‐7.4] years for normal/equivocal‐HCM cats vs 5.6 [1.8‐7.8] years for normal/equivocal‐normal/equivocal cats, *P* = .8). At baseline, cats that developed HCM (normal/equivocal‐HCM) had a higher prevalence of heart murmurs than those that did not develop HCM (68% vs 33%, *P* = .01), with thicker LV walls (*P* < .001), smaller LVIDd (*P* = .04) and a lower LAFS% (*P* = .007).

**TABLE 4:** Baseline and follow‐up characteristics of cats developing hypertrophic cardiomyopathy and cats remaining free of hypertrophic cardiomyopathy

	Normal/equivocal‐HCM	Normal/equivocal‐normal/equivocal	*P* value
At baseline			
Demographics			
Number (%)	19 (23)	64 (77)	
Age (y)	2.5 [0.5‐12.5]	2.5 [0.1‐13]	.5
Sex			
Male (%)	9 (47; n = 19)	29 (46; n = 63)	.9
Weight (kg)	3.8 [2.2‐7.9] (n = 17)	3.4 [1.3‐5.9] (n = 55)	.3
BCS (/9)	5 [4‐8] (n = 15)	5 [4‐6] (n = 54)	.4
Murmur (%)	**13 (68)**	21 (33)	**.01**
Murmur grade	3 [1‐3]	2 [1‐3]	.07
BP (systolic)	114 [100‐167] (n = 14)	120 [100‐161] (n = 51)	.8
Heart rate (bpm)	**228** (208‐248; n = 18)	205 (196‐214; n = 61)	**.02**
Echocardiography			
LVWT (mm)	**5.4** [4.0‐5.9]	4.4 [2.8‐5.8]	**<.001**
IVSd (mm)	**5.3** [4.0‐5.9]	4.3 [2.8‐5.8]	**<.001**
LVFWd (mm)	**4.7** (4.4‐5.0)	4.1 (4.0‐4.3)	**<.001**
LVIDd (mm)	**13.7** [10.2‐16.9]	14.8 [12.4‐21.1] (n = 58)	**.04**
LAD (mm)	13.8 (12.9‐14.7; n = 17)	13.3 (12.9‐13.8; n = 56)	.3
LA/Ao	1.3 [1.1‐1.5]	1.4 [1.0‐1.7] (n = 61)	.4
LAFS%	**24.5** (22‐27; n = 18)	28.3 (26.9‐29.7; n = 51)	**.01**
LVFS%	**40.6** (35.9‐45.4; n = 10)	35.9 (34.1‐37.7; n = 46)	**.03**
At follow‐up			
Demographics			
Follow‐up (y)	5.4 [1.2‐7.4]	5.6 [1.8‐7.8]	.8
Age (y)	8.7 (7.7‐9.9)	8.0 (7.3‐8.6)	.3
Weight (kg)	4.7 (4.2‐5.3; n = 14)	4.7 (4.4‐5.0; n = 51)	1.0
BCS (/9)	5 [3‐7] (n = 17)	5 [2‐8] (n = 62)	.7
Murmur (%)	**15 (79)**	24 (39; n = 62)	**.002**
Murmur grade	3 [1‐4]	2 [1‐4]	.07
BP (systolic)	135 (123‐147; n = 14)	136 (131‐142; n = 41)	.8
Heart rate (bpm)	**209** (190‐228; n = 15)	183 (173‐192; n = 40)	**.01**
Echocardiography			
LVWT (mm)	**6.4** [6‐8.8]	4.8 [3.5‐5.9]	**<.001**
IVSd (mm)	**6.2** [5.6‐7.6]	4.8 [3.5‐5.9]	**<.001**
LVFWd (mm)	**5.4** [3.9‐8.8]	4.4 [3‐5.8]	**<.001**
LVIDd (mm)	15.5 (14.4‐16.6)	15.6 (15.2‐16)	.9
LAD (mm)	**17.2** (15.7‐18.7; n = 18)	14.9 (14.5‐15.3; n = 62)	**<.001**
LA/Ao	**1.6** [1.2‐2.5]	1.4 [1.2‐1.7]	**.02**
LAFS%	**22.1** (18.5‐25.7)	27.5 (26.4‐28.5; n = 61)	**<.001**
LVFS%	39.2 (26.4‐52; n = 4)	43.7 (41.4‐46; n = 46)	.3

*Note*: Comparisons between groups were performed by Mann‐Whitney *U*‐test or students' independent *t*‐test and Pearson chi‐squared or Fisher's exact for continuous and categorical variables, respectively. Statistically significant differences (*P* < .05) are highlighted in bold.

Abbreviations: see Table 1.

**FIGURE 3:**
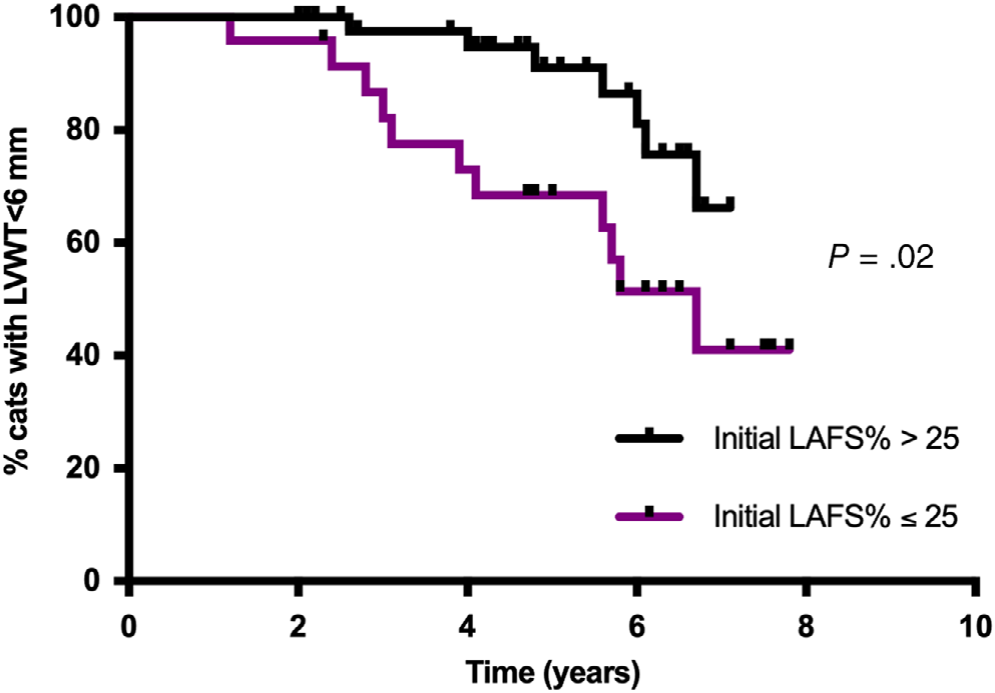
Kaplan‐Meier curve showing percentage of cats developing HCM (LVWT ≥6 mm) over time according to initial left atrial fractional shortening (LAFS%)

**FIGURE 4:**
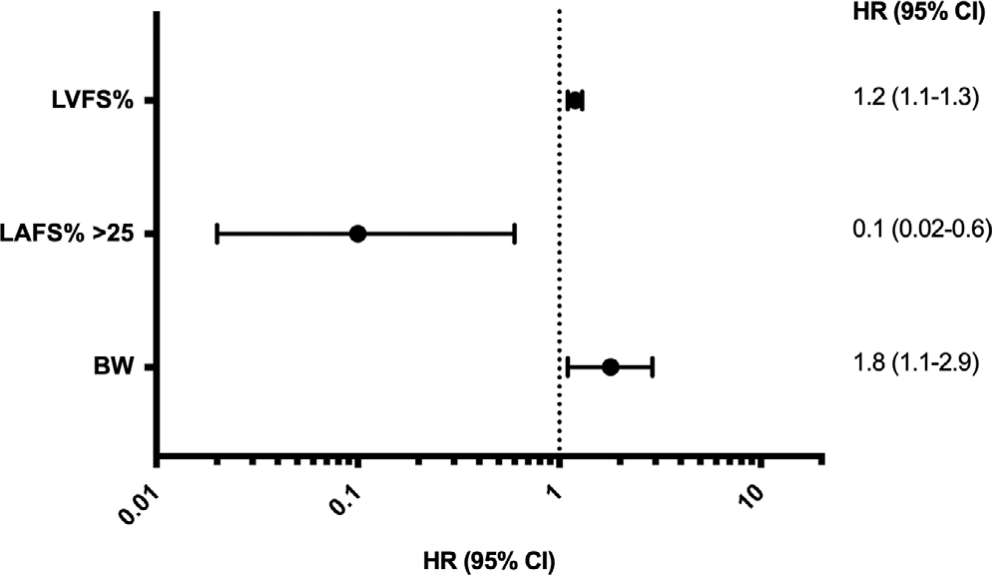
Forest plot of variables independently associated with development of HCM (LVWT ≥6 mm) in the multivariable Cox's proportional hazard analyses. Left ventricular fractional shortening (LVFS%) for a unit increase of 1%; body weight (BW) for a unit increase of 1 kg

Univariable baseline predictors of progression from normal/equivocal to HCM (LVWT ≥6 mm) included lower LAFS% (cats with an initial LAFS% ≤25%), higher LVFS% and smaller LVIDd. Cats with a LAFS% >25 at baseline were 91% less likely to develop HCM (Figure 3). In multivariable analysis, initial LAFS% ≤25%, higher LVFS% and higher BW at baseline were independently associated with increased hazard of developing HCM (Figure 4). Graphical and statistical analysis showed no violation of the proportional hazard assumptions, there was no multicollinearity, and the multivariable model was a good fit to the data.

When HCM was defined as LVWT ≥5.5 mm, none of the assessed variables at baseline was associated with a greater risk of HCM on Cox proportional hazard analyses, LAFS% was lower at baseline in cats that later developed HCM but not significantly (*P* = .05).

### 3.4 Cats with SAM

#### 3.4.1 HCM with SAM (HCM + SAM)

The proportion of cats with HCM that had SAM was similar at baseline 7/21 (33%) and follow‐up 6/21 (29%), *P* = .7, although changes were seen in individual cats. Of the 7 cats with HCM + SAM at baseline, 4 did not have SAM documented at follow‐up, although 3 other cats with HCM had SAM at follow‐up.

#### 3.4.2 Equivocal cats with SAM

Three cats had SAM with normal LVWT [5.2‐5.4] mm at baseline and were followed over 2.3‐6.1 years; 2/3 developed HCM (LVWT 7.4 and 8.8 mm) and 1 had a LVWT of 5.8 mm on follow‐up. The latter had the shortest follow‐up period (2.3 years). One cat with SAM and LVWT of 5.6 mm at baseline developed HCM + SAM (LVWT 7.3 mm) 6.7 years after first scan. All these cats had an increase in LA size over time (LA/Ao 1.5 [1.2‐1.5] vs 1.7 [1.3‐2.0] at follow‐up).

By the final follow‐up there were 40/107 (37.4%) cats with HCM; mean age at diagnosis was 7.3 (6.3‐8.4) years, 23/40 (58%) cats were male, and 16/40 (40%) cats had SAM. Among cats with HCM, those with and without SAM were similar in age: 10.3 (9.2‐11.4) years vs 9.2 (7.8‐10.6) years respectively, *P* = .2.

### 3.5 Cats that remained free of HCM during the study (normal/equivocal‐normal/equivocal)

With a median follow‐up time of 5.6 years, characteristics of the 64 cats with a normal/equivocal cardiac phenotype throughout are summarized in Table 5. Weight, body condition score, systolic blood pressure, LVWT, LVIDd and LA size increased over time.

**TABLE 5:** Baseline and follow‐up characteristics of 64 cats with normal cardiac phenotype over the study period

	N	Baseline	Follow‐up	*P* value
Demographics				
Age (y)		2.5 [0.1‐13]	7.8 [3.0‐15.6]	–
Weight (kg)	44	3.6 (3.3‐3.8)	**4.7** (4.4‐5.0)	**<.001**
BCS (/9)	52	5 [4‐6]	**5 [2‐8]**	**<.001**
Murmur (%)	61	20 (33)	23 (38)	.7
Blood pressure	34	119 [101‐161]	**136** [110‐180]	**<.001**
Heart rate (bpm)	37	204 (191‐216)	**182** (172‐192)	**.01**
Echocardiography				
LVWT (mm)		4.4 (4.2‐4.6)	**4.8** (4.7‐5.0)	**<.001**
IVSd (mm)		4.3 (4.1‐4.5)	**4.7** (4.6‐4.9)	**<.001**
LVFWd (mm)		4.1 (4.0‐4.3)	**4.4** (4.3‐4.6)	**.01**
LVIDd (mm)	58	13.9 (13.1‐14.6)	**15.1**(14.1‐16.0)	**.002**
LAD (mm)	55	13.3 (12.8‐13.7)	**15.0** (14.7‐15.4)	**<.001**
LA/Ao	61	1.4 [1.0‐1.7]	**1.4** [1.2‐1.7]	**<.001**
LAFS%	50	28.4 (27‐29.8)	27.5 (26.3‐28.7)	.3
LVFS%	32	34 [27‐51]	**44.4** [26‐57]	**<.001**

*Note*: Comparisons between baseline and follow‐up were performed by Wilcoxon signed‐rank test or paired *t*‐test for nonnormally and normally distributed continuous variables, respectively. McNemar's test was used to compare murmur prevalence.

Abbreviations: See Table 1.

### 3.6 Change in LVWT over time in cats with LVWT <6 mm at baseline

**FIGURE 5:**
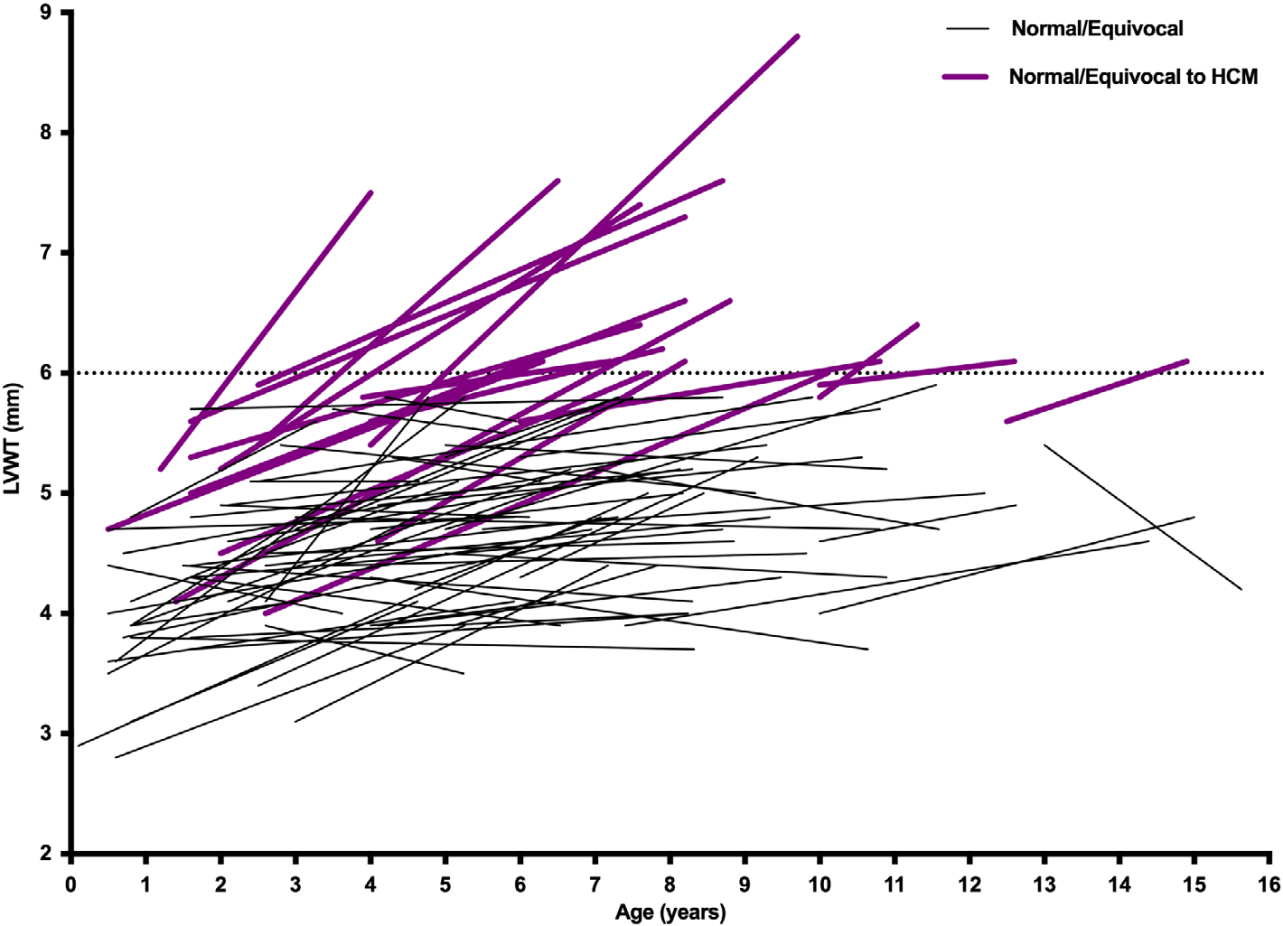
Change in left ventricular wall thickness (LVWT) over time in individual cats that were normal or equivocal at baseline. Cats that remained normal or equivocal are shown in black, and cats that developed hypertrophic cardiomyopathy (HCM) are shown in purple

In 67/83 (81%) cats the LVWT increased over the study period (Figure 5). There was no association between %ΔLVWT/year and age at baseline, BW change or systolic blood pressure change over time.

### 3.7 Cross‐sectional (CatScan I) vs longitudinal (CatScan II) study samples

Baseline age distribution was similar in the cross‐sectional (CatScan I) and longitudinal (CatScan II) study samples, with no difference in the proportion of males (43.3% in CatScan I vs 50.5% in CatScan II, *P* = .14). Cats enrolled in the longitudinal study had a higher prevalence of heart murmurs at baseline (40.8% in CatScan I vs 51% in CatScan II, *P* = .03). The baseline prevalence of HCM in the cross‐sectional and longitudinal study samples was similar (14.7% vs 19.6% respectively, *P* = .15), as was the proportion of HCM + SAM cats (35.7% vs 33.3% respectively, *P* = .8). There was a higher prevalence of cats equivocal for cardiomyopathy in the longitudinal study sample (4.0% in CatScan I vs 16.8% in CatScan II, *P* < .001).

### 3.8 Outcome in cats with HCM

Thirty‐four cats with HCM had a median follow‐up of 3.6 [0.7‐8.6] years. Cardiovascular events were observed in 7/34 (20.6%) cats with HCM. Two cats (2/34 cats, 5.9%) had CHF, 2/34 (5.9%) cats had ATE and 1/34 (2.9%) cat had CHF and ATE. Sudden death occurred in 2/34 (5.9%) cats. Incidence proportion was 7 cardiovascular events per 34 cats with HCM per 4.2 years, 20.6% per 4 years. The incidence of CHF + ATE was 34.7 events per 1000 cat years, CHF was 20.8 events per 1000 cat years, ATE was 20.8 events per 1000 cat years and SD was 13.9 events per 1000 cat years.

### 3.9 Echocardiographic measurement variability

Intraclass correlation coefficient (ICC) was 0.90 and coefficient of variation (%CV) was 5% for both LVWT and LA%FS indicating excellent intraobserver agreement and low measurement variability.

## 4 DISCUSSION

In this study, we followed a study sample of cats that were originally screened at rehoming centers in the UK with serial echocardiographic examinations over 5.6 [1.2‐9.2] years. This study sample was not referred to a specialist center and also included normal cats, providing an opportunity to document the development of HCM. The main findings of our study were: (1) 23% of cats developed HCM during the study; (2) predictors of greater risk of HCM at follow‐up included lower LAFS%, higher LVFS% and higher BW; (3) 21% of cats with HCM had cardiovascular events (ATE, CHF or SD); (4) LVWT increased over time in most cats; (5) the proportion of cats with HCM without SAM (nonobstructive HCM) was higher than in most previous reports of subclinical HCM.

In this study, 40/107 (37.4%) cats had HCM at the final follow‐up. The prevalence of subclinical HCM in cats not referred for suspected heart disease is 14.6% to 34%, increasing with age.^26^, ^27^ The higher prevalence could be related to the older age of our study sample.^17^, ^28^ The results confirm that the prevalence of HCM in cats appears to be high in the geographic region, environment, and genetic pool where this study took place.

In people, changes in LV function and geometry have been identified before the development of overt HCM (prephenotypic HCM), including increased LV ejection fraction,^14^, ^16^, ^29^ impaired LA function,^30^ diastolic dysfunction,^16^, ^31^, ^32^ smaller LV diameters and volumes,^14^ ECG changes,^16^, ^33^ increased mitral valve leaflet length,^16^, ^34^, ^35^ higher N‐terminal pro B‐type natriuretic peptide,^16^ and a profibrotic state.^36^, ^37^ Prephenotypic changes in cats include diastolic dysfunction^38^ and elongated anterior mitral valve leaflets.^39^

We assessed baseline factors in cats that subsequently developed HCM to look for predictors of HCM. Multivariable analysis showed that lower LAFS%, higher LVFS%, and higher BW independently predicted an increased hazard of developing HCM.

Cats with a baseline LAFS% >25% were much less likely to develop HCM, and reduced LA function has been suggested as an early phenotypic change in people.^30^ It has been speculated in humans that LA dysfunction might reflect early myocardial fibrosis,^30^, ^40^ which could be a marker for early HCM.^40^ A recent study showed that LAFS% in cats with subclinical HCM was correlated with LV extracellular volume, a measure of interstitial fibrosis, but not with LVWT.^41^ Abnormal collagen metabolism has been also suggested in Ragdoll cats carrying *MYBPC3* R820W mutation without LVH,^42^ and interstitial fibrosis has been described on histopathology in cats diagnosed antemortem with mild subclinical HCM.^43^ Thus, our findings of LA dysfunction might, as in humans, reflect abnormal myocardial properties that precede overt HCM. LA dysfunction is recognized to be an important prognostic factor in overt HCM,^44^, ^45^ and we suggest that LA dysfunction might be an important abnormality even before manifestation of the HCM phenotype.

Cats with higher LVFS% were also more likely to develop HCM. In people, higher LV ejection fraction has been documented before the development of HCM.^14^, ^16^, ^29^, ^46^ Hyperdynamic LV function detected by speckle‐tracking echocardiography and cardiac magnetic resonance imaging is present in prephenotypic HCM.^47^, ^48^ It has been proposed that a hyperdynamic state in HCM might reflect intrinsic myocardial properties associated with sarcomere mutations^29^ that increase sarcomere power output.^49^ Targeting sarcomere hypercontractility with a cardiac myosin inhibitor^49^, ^50^, ^51^ halts the development and progression of HCM,^49^ suggesting enhanced sarcomere function might be an important mechanism in the development of HCM (LVH). The higher LV%FS identified in our study might parallel the prephenotypic hyperdynamic systolic function described in humans. Alternatively, the thicker LV walls and smaller LVIDd at baseline (Table 4) in the cats developing HCM might have resulted in higher LVFS% due to lower afterload,^52^, ^53^, ^54^ rather than a true hypercontractile state.

Heavier cats at baseline had a greater risk of developing HCM, but the LVWT change over time was not associated with changes in BW. Higher BW is associated with increased LVWT in cats,^8^, ^17^, ^42^, ^55^, ^56^ so this might explain why BW was a risk factor for HCM. Alternatively heavier cats might have a greater risk of HCM associated with a yet unidentified epigenetic or environmental factor. The cardiac effects of obesity in cats are not clear‐cut with some studies showing an association between LVH and obesity,^17^, ^57^ while others show none.^56^, ^58^ In our study, body condition score was not associated with a higher risk of HCM, so size (BW) rather than obesity appeared to be responsible. Allometric scaling to normalize linear echocardiographic variables to BW have been described in cats.^55^, ^59^, ^60^ We did not normalize LVWT to BW in this study, but this could have helped to determine whether size/BW was a truly independent risk factor for HCM.

Cats that developed HCM during our study had a higher HR at baseline than cats that remained normal (Table 4), but HR was not a predictor of HCM in the multivariable analysis. The reasons for the higher HR are not clear (Tables 3, 4, 5). Although higher HR can cause pseudohypertrophy,^61^ it has not been shown to cause LVWT ≥6 mm.^61^ Cats that developed HCM (LVWT ≥6 mm) had LA dilation, higher N‐terminal pro B‐type natriuretic peptide, or developed cardiovascular events, or both, so higher HR alone is not likely to be the cause of the LV wall thickening.

The REVEAL study of cats with subclinical HCM (stage B) described HCM + SAM in 57% of cats,^8^ vs 40% in our study. This suggests that nonobstructive HCM might be more prevalent in nonreferral study samples. Asymptomatic cats without a murmur are unlikely to be referred for echocardiography, and thus nonobstructive HCM might be underrepresented in referral‐based studies. Larger studies in a general cat study group would be required to explore this finding further.

Four cats with normal‐equivocal LVWT and SAM at baseline showed an increased LVWT over time, associated with increasing LA size, suggesting that SAM might be an early phenotypic (pre‐HCM) change. However, LVH could also potentially develop as a consequence of pressure overload associated with LVOTO. Elongated mitral valve leaflets are associated with SAM and have been suggested as an early morphological marker of HCM.^16^, ^34^, ^35^, ^39^

Two cats with HCM developed regional thin and hypokinetic myocardial segments, but otherwise cats with HCM had stable LVH over time, and no cats showed reverse remodeling.

Cardiovascular events (CHF, ATE, or SD) were observed in 20.6% of cats with HCM over a mean study period of 4.2 years. Either CHF, ATE, or both was observed in 14.7% of cats with HCM in the present study. The overall incidence of cardiovascular events is 30%.^8^ The percentage of cases and incidence of ATE and SD in our study was similar to REVEAL (REVEAL 26.6 ATE events per 1000 cat years vs CatScan II 20.8 ATE events per 1000 cat years; REVEAL 5.0 SD events per 1000 cat years vs CatScan II 13.9 SD events per 1000 cat years) and only CHF cases were more frequently described in the latter (incidence rate 57.6 CHF events per 1000 cat years vs 20.8 events per 1000 cat years in our study). The difference in CHF incidence between the studies might be caused by a longer follow‐up period in REVEAL, as all enrolled cats in that study had at least a 5‐year follow‐up.^8^ A smaller prospective study in a primary care setting also showed that 30% of cats with subclinical HCM suffered a cardiovascular event over a 3‐year follow‐up period.^62^ These 2 studies suggest that around one third of cats with subclinical HCM in either a referral or primary care setting develop a cardiovascular event,^8^, ^62^ with our study showing a similar incidence.

An increase in LVWT over time was seen in most cats, whether or not they developed HCM. The increase in LVWT was not associated with age at baseline, BW, or systolic blood pressure change during the study. There is a progressive increase in LVWT in human patients with HCM.^63^

A large number of cats were lost to follow‐up, as might be expected in a large longitudinal study following cats after rehoming. At baseline, longitudinal and cross‐sectional study samples had similar demographic characteristics, and the prevalence of HCM was similar. However, there was a higher prevalence of cats with heart murmurs and cats with equivocal cardiomyopathy in the longitudinal study sample. This might reflect a selection bias, where owners of cats known to have a heart murmur and, or with a suspected cardiomyopathy, or both, were more likely to enroll their cats into the study or to participate in the follow‐up examinations.

We defined HCM as LVWT ≥6 mm, but there is no single cut‐off value for LVWT that will reliably differentiate cats with and without HCM.^18^, ^55^, ^64^ We used a cut‐off of 6 mm to err on the side of high specificity for identifying LVH. Our findings might have been different if we had used a lower cut‐off, or normalized LVWT to BW,^55^, ^59^, ^60^ but there is currently no consensus on cut‐off values for defining HCM using allometric scaling. We repeated the Cox regression analysis using a 5.5 mm cut‐off to diagnose HCM, and none of the baseline variables were associated with a higher hazard of HCM, although LAFS% was also lower at baseline in cats that later developed HCM albeit not significantly (*P* = .05).

Our study had some limitations. We evaluated a sample of cats originating from 2 rehoming centers, thereby avoiding potential referral biases. Nevertheless, other types of bias might have been present, such as selection or survivor bias. Owners of cats known to have heart disease or a heart murmur might have been more likely to enroll their cats in the longitudinal arm of the study (selection bias). Additionally, the large number of cats lost to follow‐up could have skewed the data, impacting the results (survivor bias).^65^ Cats lost to follow‐up (Table 1) were not included in the outcome analysis, and therefore our cardiovascular event rate and death rate might be underestimated.

In the present study, LVOTO was only assessed by the presence of SAM, as a LVOT gradient was not available in the majority of cats, and there is no consensus on what constitutes “severe” LVOTO in cats. SAM is a labile phenomenon and thus some cats classified as HCM without SAM might have demonstrated SAM under other situations (eg, higher sympathetic tone), so this finding should be interpreted with caution.

In conclusion, in a study sample of cats from rehoming centers not referred for suspected heart disease 37% had HCM at follow‐up, and 21% of cats with HCM experienced cardiovascular events. Lower LA systolic function preceded subsequent development of overt HCM.

## CONFLICT OF INTEREST DECLARATION

Authors declare no conflict of interest.

## OFF‐LABEL ANTIMICROBIAL DECLARATION

Authors declare no off‐label use of antimicrobials.

## INSTITUTIONAL ANIMAL CARE AND USE COMMITTEE (IACUC) OR OTHER APPROVAL DECLARATION

Approved by the Clinical Research Ethical Review Board of the Royal Veterinary College (URN 2010‐1004).

## HUMAN ETHICS APPROVAL DECLARATION

Authors declare human ethics approval was not needed for this study.
